# 缓解后FIT状态急性髓系白血病接受持续VA与中大剂量阿糖胞苷巩固治疗疗效比较

**DOI:** 10.3760/cma.j.cn121090-20240529-00198

**Published:** 2025-04

**Authors:** 立 孙, 朋朋 张, 思楣 任, 楠 周, 丽媛 李, 真真 王, 伟广 崔, 帆 杨, 建民 罗, 琳 杨

**Affiliations:** 1 河北医科大学第二医院血液科，河北省血液病学重点实验室，石家庄 050000 Department of Hematology, the Second Hospital of Hebei Medical University, Key Laboratory of Hematology, Shijiazhuang 050000, China; 2 北京医院，国家老年医学中心，国家卫生健康委临床检验中心，中国医学科学院老年医学研究院，北京 100730 Beijing Hospital, Institute of Geriatric Medicine, National Center for Clinical Laboratories, National Center of Gerontology, Chinese Academy of Medical Sciences, Beijing 100730, China

**Keywords:** 白血病，髓系，急性, 老年人, 维奈克拉, 阿糖胞苷, 缓解后治疗, Leukemia, myeloid, acute, Aged, Venetoclax, Cytarabine, Post-remission treatment

## Abstract

**目的:**

比较维奈克拉联合阿扎胞苷（VA）持续化疗和中大剂量阿糖胞苷（I/HDAC）巩固强化治疗对缓解后转FIT（Fit for standard chemotherapy）状态急性髓系白血病（AML）的疗效及安全性。

**方法:**

收集2020年8月至2024年1月在河北医科大学第二医院血液科经VA方案诱导治疗后FIT状态患者的缓解后治疗临床资料，回顾性分析VA持续化疗和I/HDAC治疗方案疗效差异，观察指标包括总生存（OS）期、无复发生存（RFS）期、无事件生存（EFS）期，以及化疗相关不良反应发生率。

**结果:**

共收集69例患者资料，其中VA方案持续治疗组46例，I/HDAC巩固强化化疗组23例。VA组中位OS、RFS、EFS期分别为26.18、24.69、20.34个月，I/HDAC组分别为34.14、30.99、28.42个月，差异均无统计学意义（*P*值均>0.05）。I/HDAC组中欧洲白血病网（ELN）低危、微小残留病（MRD）阳性、FLT3野生型和IDH1/2突变亚组的中位OS期较VA组中相应亚组延长（*P*值均<0.05）。VA组3～4级粒细胞减少、3～4级血小板减少及血流感染发生率显著低于I/HDAC组（*P*值均<0.05）。

**结论:**

对于ELN低危、MRD阳性、FLT3野生型或IDH1/2突变的缓解后FIT状态AML患者，接受I/HDAC巩固强化化疗可能较持续VA化疗OS期延长。缓解后VA持续化疗安全性优于I/HDAC巩固强化化疗。

## Abstract (en)

**Objective:**

To compare the efficacy and safety of continuous venetoclax combined azacitidine（VA）chemotherapy and intermedium/high-dose cytarabine（I/HDAC）consolidation in patients with acute myeloid leukemia（AML）fit for standard chemotherapy（transform from UNFIT）.

**Methods:**

Clinical data of patients who were fit for standard chemotherapy were collected among those with AML who underwent VA induction in the Department of Hematology, the Second Hospital of Hebei Medical University. The overall survival（OS）, relapse-free survival（RFS）, event-free survival（EFS）, and incidence of adverse events were analyzed retrospectively.

**Results:**

This study enrolled 69 patients, consisting of 46 cases in the VA group and 23 cases in the I/HDAC group. We revealed the following. ① The median OS, RFS,EFS were 26.18, 24.69, 20.34 months in the VA group, and 34.14, 30.99, 28.42 months in the I/HDAC group, respectively, with no statistically significant difference（all *P*>0.05）. Median OS of patients who underwent I/HDAC consolidation with European Leukemia Net（ELN）favorable-risk, positive measurable residual disease（MRD）, wild type FLT3, or IDH1/2 mutation was significantly longer than those who received VA（*P*<0.05）. ②Adverse events rate of grade 3–4 neutropenia, grade 3–4 thrombocytopenia, and bacteremia were significantly lower in the VA group than in the I/HDAC group（*P*<0.05）.

**Conclusion:**

I/HDAC consolidation was more likely to help get survival benefits for patients with ELN favorable-risk, positive MRD, wild type FLT3, or IDH1/2 mutation. Continuous VA chemotherapy exhibited superior safety than I/HDAC consolidation.

因老龄或存在严重非血液学合并症而不适合标准化疗（Unfit for standard chemotherapy, UNFIT）急性髓系白血病（AML）患者是AML领域的治疗难点[1]。2021年中国成人AML诊疗指南首次推荐维奈克拉联合阿扎胞苷（VA）化疗方案用于UNFIT AML的诱导治疗[2]，显著提高了UNFIT AML缓解率[3]，但缓解后治疗仍无统一方案。数据显示60岁以上高危AML患者1年内复发率可达80％[4]。

根据2023年中国成人AML诊疗指南[5]，UNFIT AML缓解后转为FIT（Fit for standard chemotherapy）状态者，低危或中高危患者均可选择中大剂量阿糖胞苷（I/HDAC）方案巩固强化化疗，或者持续VA方案化疗。哪种缓解后治疗疗效更佳目前仍有争议[6]。真实世界研究中未见此两种方案在AML缓解后治疗的疗效及安全性比较。另有新近研究显示，NPM1阳性预后良好的AML患者，应用VA方案化疗4个周期内获得分子学微小残留病（MRD）阴性者，停止治疗后2年无治疗缓解率高达88％[7]，可见在预后良好AML的缓解后治疗中，强化疗的必要性也不可一概而论。

本研究对单中心接受缓解后治疗的AML病例资料进行回顾分析，比较VA持续化疗与I/HDAC巩固强化化疗对缓解后FIT状态AML的疗效及安全性，以期为VA方案诱导达缓解且转FIT状态AML临床治疗策略的选择提供依据。

## 病例与方法

一、研究对象

纳入2020年8月至2024年1月河北医科大学第二医院血液科经≤2个疗程VA方案诱导达缓解并接受VA或I/HDAC方案巩固治疗的AML（非急性早幼粒细胞白血病）患者，剔除缓解后仍UNFIT状态者。AML的诊断按照2016版WHO造血和淋巴组织肿瘤分类标准[8]。按照FERRARA标准[9]满足以下至少一项定义为UNFIT状态：①年龄≥75岁；②存在严重心脏合并症；③存在严重肺合并症；④存在严重肾合并症；⑤存在严重肝合并症；⑥不能控制的活动性感染；⑦存在认知障碍；⑧美国东部肿瘤协作组（ECOG）评分>２分，或不适合化疗的其他合并症。按照2017欧洲白血病网（ELN）标准进行危险度分层[10]。

二、资料收集

包括年龄、性别，缓解后基线中性粒细胞值、HGB、PLT、骨髓细胞形态学、免疫分型、染色体核型、融合基因和二代测序报告，每疗程化疗前血常规、骨髓细胞形态学、MRD，以及化疗期间发生的血液学和非血液学不良反应的临床资料。本研究为回顾性研究，经过河北医科大学第二医院伦理委员会审查和批准，知情同意被豁免。

三、研究流程

本中心纳入2020年8月至2024年1月期间经≤2个疗程VA方案诱导达完全缓解（CR）/CR伴血液学不完全恢复（CRi）的患者，缓解后重新评估疾病状态，UNFIT状态AML患者继续VA方案持续化疗。缓解后FIT状态的AML患者评估其是否有条件行造血干细胞移植巩固治疗：ELN低危患者可选择自体造血干细胞移植，ELN中高危或ELN低中危但MRD阳性患者，建议行异基因造血干细胞移植（allo-HSCT）。无条件行造血干细胞移植巩固治疗者，结合患者主观意愿，选择VA方案持续化疗，或者3～4个周期I/HDAC方案化疗［此部分患者中，若患者ELN低危且MRD持续阴性，后期停止化疗并定期监测MRD；若患者ELN中高危或ELN低危但MRD阳性，后期给予阿扎胞苷（75 mg/m^2^，第1～7天）维持治疗直至疾病进展或复发］。

四、治疗方案

1. VA持续化疗方案：标准方案为阿扎胞苷75 mg/m^2^第1～7天，维奈克拉400 mg第1～28天，28 d为1个周期。若化疗间期存在造血恢复延迟（化疗后第14天中性粒细胞绝对值<1.0×10^9^/L或PLT<100×10^9^/L），将每疗程维奈克拉天数依次调整为21、14或7 d[11]–[12]。持续化疗直至疾病进展或复发。

2. I/HDAC化疗方案：对于≥60岁且<75岁患者，给予3～4个周期中剂量阿糖胞苷方案（IDAC，1～1.5 g/m^2^，每12 h 1次，第1～3天）化疗，持续静脉滴注大于3 h；对于<60岁患者，给予3～4个周期大剂量阿糖胞苷方案（HDAC，2～3 g/m^2^，每12 h 1次，第1～3天）化疗，持续静脉滴注大于3 h。

3. 分子靶向药物的联合应用：伴FLT3-ITD/TKD突变患者，缓解后治疗联合吉瑞替尼80 mg或120 mg，每日1次；伴IDH1突变患者，缓解后治疗联合艾伏尼布500 mg，每日1次；伴IDH2突变患者，缓解后治疗联合恩西地平100 mg，每日1次。

4. 支持治疗：化疗期间监测血液学及非血液学不良反应，及时给予止吐、成分血输注、细胞因子刺激造血（G-CSF、IL-11）及抗感染等支持治疗。

5. 骨髓抑制期给予环境保护，按中国中性粒细胞缺乏伴发热指导原则进行预防、控制。对发热患者行血液培养和药敏试验，完善C反应蛋白、降钙素原和G/GM试验检测。

五、疗效及不良反应判定

依据2017ELN指南行疗效评价[10]，流式细胞术检测骨髓异常的髓系原始细胞，若异常髓系原始细胞≤0.1％，评定为MRD阴性。依据2017NCI肿瘤治疗常见不良事件评价标准（CTCAE）5.0版[13]对不良反应判定。计算总生存（OS）期（获得缓解至死亡或末次随访）、无复发生存（RFS）期（达无白血病状态至死亡、复发或末次随访）和无事件生存（EFS）期（获得缓解至治疗失败、复发或死亡）。

六、随访

回顾患者门诊和入院病历，电话回访，记录生存、复发及并发症情况，随访截至2024年2月29日。

七、统计学处理

以SPSS 27.0软件行统计分析，计量数据采用Shapiro-Wilk正态检验，服从正态分布资料以*x*±*s*表示，使用非配对样本*t*检验进行差异分析。不符合正态分布资料用*M*（范围）表示，采用非配对样本Wilcoxon检验分析。采用Kaplan-Meier法进行生存分析，采用Log-rank检验法进行组间比较。其中年龄亚组疗效应用STATA 15.0软件的STEPP方法进行多重比较。*P*<0.05为差异有统计学意义。

## 结果

一、患者一般临床特征

共收集69例病例资料，VA组46例，I/HDAC组23例。患者一般特征详见表1。两组患者年龄、性别、ELN危险度、MRD阴性率、基线中性粒细胞值、HGB、PLT及诊断时UNFIT因素方面差异均无统计学意义。经allo-HSCT巩固治疗者4例，其中VA组3例（6.5％），I/HDAC组1例（4.3％），差异无统计学意义。VA组有11例（23.9％）继发AML病例，I/HDAC组无继发AML病例，差异有统计学意义。考虑该差异会影响两种方案疗效比较，在两组疗效比较前剔除11例继发AML病例。

**表1:** 69例缓解后FIT状态急性髓系白血病患者一般临床特征［例（％）］

特征	VA组（46例）	中大剂量阿糖胞苷组（23例）
年龄［岁，*M*（范围）］	65（20～73）	57（27～67）
性别［例（％）］		
男	20（43.5）	7（30.4）
女	26（56.5）	16（69.6）
疾病类型［例（％）］		
原发	35（76.1）	23（100.0）^a^
继发	11（23.9）	0（0）
ELN危险度分层［例（％）］		
低危	6（13.0）	7（30.4）
中危	24（52.2）	8（34.8）
高危	16（34.8）	8（34.8）
MRD［例（％）］		
阴性	37（80.9）	21（69.9）
阳性	9（19.1）	2（30.4）
中性粒细胞绝对计数（×10^9^/L，*x*±*s*）	5.6±3.0	6.8±3.6
HGB（g/L，*x*±*s*）	94.6±13.9	89.8±22.4
PLT（×10^9^/L，*x*±*s*）	171.2±59.4	129.3±35.1
基因突变［例（％）］		
IDH1/2	14（30.4）	8（34.7）
FLT3-ITD/TKD	14（30.4）	7（30.4）
DNMT3A	18（39.1）	8（34.8）
NPM1	10（21.7）	9（39.1）
K/N-RAS	7（15.2）	5（21.7）
TET2	7（15.2）	5（21.7）
ASXL1	7（15.2）	4（17.4）
CEBPA-bZIP	1（2.2）	1（4.3）
RUNX1	6（13.0）	3（13.0）
TP53	10（21.7）	2（8.7）
JAK2	2（4.3）	1（4.3）
诊断时UNFIT因素［例（％）］		
活动性感染	21（45.7）	12（52.2）
ECOG≥3分	22（47.8）	10（43.5）
心脏合并症	2（4.3）	0（0）
肺合并症	0（0）	1（4.3）
肾脏合并症	1（2.2）	0（0）

**注** ^a^两组比较*P*<0.05；VA：维奈克拉联合阿扎胞苷；ELN：欧洲白血病网；MRD：微小残留病；ECOG：美国东部肿瘤协作组

二、疗效比较

1. 总体比较：VA持续化疗中位周期3（1～11）个，I/HDAC巩固强化化疗中位周期2（1～4）个。总体中位随访18.75个月，VA组中位随访17.97个月，I/HDAC组中位随访19.73个月，随访时间差异无统计学意义。截至随访终点，VA组（剔除继发AML病例后35例）生存28例（80.0％）、死亡7例（20.0％），死亡患者2例死于重症肺炎，1例死于脑出血，4例死于疾病复发；I/HDAC组生存20例（87.0％）、死亡3例（13.0％），死亡患者1例死于脓毒症，2例死于疾病复发。VA组与I/HDAC组中位OS、RFS、EFS期差异均无统计学意义（OS：26.18个月对34.14个月，*P*＝0.180；RFS：24.69个月对30.99个月，*P*＝0.346；EFS：20.34个月对28.42个月，*P*＝0.131）。

2. 亚组疗效比较：详见表2。I/HDAC组ELN低危亚组、MRD阳性亚组、FLT3野生型亚组、IDH1/2突变亚组中位OS期均较VA组延长（均*P*<0.05）；另外VA组内的亚组疗效比较显示，获得CRi亚组较获得CR亚组中位OS期明显缩短（*P*<0.05）；IDH1/2野生型亚组较IDH1/2突变亚组中位RFS期、中位EFS期显著缩短（*P*<0.05）。

**表2:** 急性髓系白血病不同亚组缓解后应用VA方案与I/HDAC方案巩固治疗的疗效比较（月）

组别	VA组				I/HDAC组			
	例数	中位OS	中位RFS	中位EFS	例数	中位OS	中位RFS	中位EFS
ELN分层								
低危	6	22.73	22.56	18.04	7	未达到^a^	未达到^a^	未达到^a^
高中危	29	25.57	23.98	20.24	16	29.72	25.39	22.17
MRD								
阴性	29	25.72	24.20	20.21	21	33.77	31.80	28.95
阳性	6	20.26	19.24	17.03	2	未达到^a^	22.17	22.17
缓解状态								
CR	23	27.52	23.98	21.40	17	36.33	29.64	27.87
CRi	12	17.17^b^	18.36	14.66	6	30.39	32.31	28.74
FLT3突变								
是	12	27.37	25.69	21.63	7	26.88	21.34	18.76
否	23	22.04	20.82	17.56	16	36.37^a^	34.17	32.24^a^
IDH1/2突变								
是	13	26.16	27.72	24.15	8	未达到^a^	32.38	32.38
否	22	23.65	20.67^b^	16.63^b^	15	31.35	29.11	25.21

**注** VA：维奈克拉联合阿扎胞苷；I/HDAC：中大剂量阿糖胞苷；ELN：欧洲白血病网；MRD：微小残留病；CR：完全缓解；CRi：CR伴血液学不完全恢复；OS：总生存期；RFS：无复发生存期；EFS：无事件生存期；^a^不同方案组间比较*P*<0.05；^b^VA组内比较*P*<0.05

3. 不同维奈克拉应用时间组疗效比较：在应用VA持续化疗的46例AML缓解后患者中，根据患者疗程中应用维奈克拉的天数进行分组，28 d 13例，21 d 5例，14 d 23例，7 d 5例，四组的中位OS、RFS、EFS期差异均无统计学意义（均*P*>0.05）。

三、不良反应

1. 血液学不良反应：VA组的3～4级中性粒细胞减少发生率显著低于I/HDAC组（17.4％对73.9％，*P*<0.05），3～4级血小板减少发生率亦显著低于I/HDAC组（15.2％对65.2％，*P*<0.05）。

2. 非血液学不良反应：最常见的为感染，其次为消化道反应。VA组血流感染发生率显著低于I/HDAC组（37.0％对73.9％，*P*<0.05）。

## 讨论

诱导达缓解的AML仍有10^9^左右肿瘤负荷[14]，缓解后治疗极为重要。本回顾性研究结果提示，ELN危险度、MRD以及分子学突变情况对缓解后治疗选择有指导意义：ELN低危患者经I/HDAC强化疗可能获益更多，与目前主流观点一致[14]；MRD阳性患者应用I/HDAC强化疗较持续VA方案可能改善OS，缓解后MRD的价值已得到公认[6]，[15]，但仍有50％以上的复发出现在MRD阴性患者[15]，这部分患者需要关注其分子学突变。

IDH是异柠檬酸脱氢酶编码基因，突变后使DNA和组蛋白发生超甲基化，细胞髓系分化受阻。研究显示IDH1/2突变患者对VA方案诱导化疗敏感[12]，[16]，我们发现缓解后阶段IDH1/2突变患者应用阿糖胞苷化疗较VA持续化疗OS更优。IDH1/2突变可能是AML患者阿糖胞苷敏感性的重要标志，欧洲和非洲人群IDH2突变均与阿糖胞苷高敏感性有关[17]。IDH突变预后可能取决于伴随突变[18]，Green等[19]统计1 333例AML患者发现IDH突变患者预后与FLT3突变相关，IDH1突变是FLT3野生型患者复发的独立危险因素，IDH1突变同时FLT3突变患者复发率低。有研究显示维奈克拉可能改善FLT3突变AML患者预后[20]，FLT3突变是影响I/HDAC方案巩固治疗AML患者复发的预后不良因素[21]，这些结果与本研究一致。FLT3及IDH1/2突变均是AML的常见突变[6]，其在AML缓解后治疗选择中的意义有待更大样本量的研究证实。

VA诱导缓解后治疗策略目前尚无大规模临床研究。在VIALE-C临床研究中，对于新诊断UNFIT状态AML患者，维奈克拉与小剂量阿糖胞苷化疗（LDAC）联合较单纯化疗显著延长OS[22]。Pei等[23]研究发现部分AML患者体内同时存在经典低分化白血病干细胞和单核性白血病干细胞，后者对嘌呤代谢显著依赖，在小鼠模型中VA化疗选择性清除经典低分化白血病干细胞，而VA与克拉屈滨联合能同时清除两种白血病干细胞。这些研究均提示传统化疗药物与维奈克拉联合应用有望改善AML患者疗效。余国攀等[24]分析了26例VA诱导转FIT状态的缓解后AML患者，缓解后调整为强化疗（其中18例行allo-HSCT）较持续VA化疗复发率降低、中位OS期延长，ELN高危组患者更获益于其强化疗调整。

AML由于其疾病异质性大，维持治疗复杂，需充分评估治疗风险/受益比。对于复杂核型、存在TP53突变、复发/难治或移植后MRD持续阳性的高危AML，维持治疗十分必要。然而预后良好的AML，维持治疗的获益尚不确定。越来越多AML患者缓解后持续应用VA化疗，如何界定其维持治疗界限？MD Anderson癌症中心认为，持续低强度化疗情况下，若获得MRD阴性或稳定低水平MRD的缓解，原化疗剂量或频率减少50％以上的方案可界定为维持治疗[25]。本研究中未观察到持续VA化疗的累积毒性，其安全性显著优于I/HDAC强化疗。

本研究提示，VA诱导缓解后FIT状态的AML患者中ELN低危、MRD阳性者，尤其存在IDH1/2突变、FLT3野生型患者，接受I/HDAC强化疗可能获得生存获益。本研究存在纳入病例数量少，患者异质性大以及随访时间较短等不足，可能影响结论可靠性，我们会在今后临床实践中进一步验证和补充，探索更有效且安全的缓解后治疗方案。
